# Saving Lives in Thoracic Surgery: Balancing Oncological Radicality and Functional Preservation, Transitioning from Standard Pneumonectomy to Targeted Sublobar Resection

**DOI:** 10.3390/cancers16040819

**Published:** 2024-02-18

**Authors:** Takashi Eguchi, Hirotaka Kumeda, Kentaro Miura, Kazutoshi Hamanaka, Kimihiro Shimizu

**Affiliations:** Division of General Thoracic Surgery, Department of Surgery, Shinshu University School of Medicine, Matsumoto 390-8621, Japan; hkumeda@shinshu-u.ac.jp (H.K.); kmiura@shinshu-u.ac.jp (K.M.); kham@shinshu-u.ac.jp (K.H.); kmshimizu@shinshu-u.ac.jp (K.S.)

**Keywords:** thoracic surgery, pneumonectomy, sublobar resection, lung cancer, minimally invasive surgery, robot-assisted thoracic surgery, oncological radicality, pulmonary function preservation

## Abstract

**Simple Summary:**

This manuscript provides a detailed analysis of the advancements in thoracic surgery. It traces the journey from the standard pneumonectomy to the precise approach of sublobar resections. The manuscript emphasizes the emergence and acceptance of advanced techniques such as robotic-assisted surgery. It also highlights the pivotal role of preserving organ functionality while ensuring oncological radicality, which is especially important when treating early-stage lung cancer. Through a comprehensive review of contemporary surgical methods and their clinical outcomes, this paper explains the delicate balance that modern thoracic surgeons must achieve between aggressive cancer removal and preserving the quality of life. It thereby contributes to the ongoing refinement of the field.

**Abstract:**

This review chronicles the evolution of thoracic surgical interventions, from the standardized pneumonectomy to the precise approach of sublobar resections. It discusses the emergence and acceptance of minimally invasive and robot-assisted surgical techniques, highlighting their impact on improving outcomes beyond cancer and their influence on the surgical management of early-stage lung cancer. Evaluating historical developments alongside present methodologies, this review underscores the critical need for meticulous surgical planning and execution to optimize both oncological radicality and functional preservation. This evolution portrayed not only technical advancements but also a shift in the clinical approach towards tailored, organ-preserving methodologies, culminating in a contemporary framework promoting sublobar resections as the standard for specific patient profiles, signifying a new era of precision in thoracic surgery.

## 1. Introduction

Thoracic surgeons often encounter complex cases, exemplified by Case 1: a young, healthy non-smoker with a history of lung adenocarcinoma and bilateral, multiple subsolid nodules, presenting the dilemma of balancing thorough cancer removal with the risk of overtreatment; and Case 2: an older smoker with significant comorbidities and a newly identified lung nodule, where the risks of surgery may outweigh its benefits. These scenarios emphasize the necessity for a personalized approach, as a standard pneumonectomy or lobectomy could significantly impact the patients’ quality of life.

For the younger patient, the emphasis lies on longitudinal assessment to discern tumors truly requiring intervention, considering potential future growth and new lesions. The strategy extends beyond addressing the immediate threat, encompassing preparation for future challenges. In contrast, for the older patient with multiple health issues, the evaluation focuses on assessing the feasibility and safety of surgery, considering less invasive alternatives when surgical risks are too high. Both cases demand a nuanced approach, integrating multifactorial assessments with interdisciplinary treatment planning to optimize patient outcomes.

The main responsibility of surgeons is to save lives. While advancements in surgical techniques and technologies have significantly improved cancer treatment outcomes, it is imperative to recognize that these are not the ultimate goals, but rather the means to achieve the overarching objective of preserving lives. Striking a balance between oncological radicality and functional preservation is crucial. An excessive focus or bias toward achieving surgical completeness, adopting state-of-the-art techniques, or assessing functional risks can pose hazards by diverting attention from the holistic well-being of the patient.

In this context, considering noncancer-related outcomes alongside oncological results becomes essential. Lung cancer patients often face heightened risks of cardiovascular and respiratory diseases due to shared factors such as smoking and advancing age [1,2,3,4]. While surgery effectively addresses cancer, it can exacerbate these risks, leading to increased noncancer-related morbidity and mortality [5,6,7]. Hence, a comprehensive approach to lung cancer care must encompass both cancer- and noncancer-related outcomes to optimize patient survival and quality of life.

This paper aims to review pivotal milestones in thoracic surgery, emphasizing the mutual relationships between groundbreaking technological or scientific advancements and their subsequent impact on thoracic surgical practice. By contextualizing historical advancements in thoracic surgery and providing insights into its future trajectory, this approach aims to guide thoracic surgeons in delivering care that not only effectively treats cancer but also mitigates noncancer-related risks, thereby fulfilling the ultimate objective of preserving lives.

## 2. Importance of Considering Noncancer-Related Outcomes in Lung Cancer Care

While the primary focus in treating lung cancer often centers around oncological outcomes, it is crucial to acknowledge noncancer-related outcomes [5,6,7]. Lung cancer patients typically face elevated risks of cardiovascular and respiratory diseases due to shared risk factors such as smoking and advanced age [1,2,3,4]. Although surgery effectively targets cancer, it can exacerbate these risks, particularly in the early postoperative period, thereby increasing noncancer-related morbidity and mortality [5]. Addressing postoperative complications becomes pivotal, given their potential to escalate the risk of noncancer-related deaths [6]. Advances in surgical techniques offer promising solutions; minimally invasive surgeries (MIS) have been demonstrated to reduce noncancer-related deaths [6] by minimizing surgical trauma and hastening recovery.

Recent trials, such as JCOG0802, have highlighted segmentectomy’s superiority in overall survival rates compared with lobectomy, particularly in reducing deaths from other diseases such as respiratory and cerebrovascular diseases [7]. It is essential to note that JCOG0802 specifically targeted selected patients in clinical stage IA with peripheral ≤2 cm tumors. This emphasis on noncancer outcomes in patients with lung cancer is particularly relevant in early-stage diseases, where noncancer comorbidities significantly impact long-term outcomes. Retrospective studies have shed light on the prognostic impact of comorbidities, revealing that noncancer risks play a more significant role in these patients, who are more likely to die from causes other than their cancer [1,2,3,4]. The CALGB140503 trial, focusing on patients with peripheral ≤2 cm tumors without nodal involvement, further corroborates this by showing that sublobar resection has been associated with lower 30-day and 90-day mortality rates (0.6% and 1.2%, respectively) compared with a lobectomy (1.1% and 1.7%, respectively), especially when performed through MIS, which constituted 80% of the procedures in the study [8]. Therefore, a comprehensive approach to lung cancer care should encompass considerations of both cancer-related and noncancer-related outcomes to enhance patient survival and elevate their quality of life.

**Figure 1:**
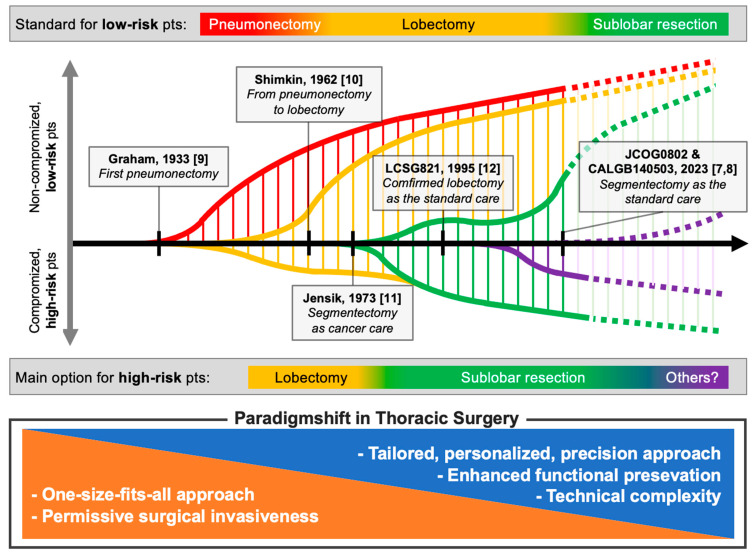
Evolving standards and paradigms in lung cancer surgery [7,8,9,10,11,12]. Legends: The red, yellow, and green lines indicate pneumonectomy, lobectomy, and sublobar resection, respectively, as surgical techniques. The purple line signifies non-surgical treatments such as stereotactic body radiotherapy. The length of the vertical lines, represented by narrow lines in corresponding colours, visually indicates the relative size of patient populations undergoing each procedure over time; this does not denote exact numerical values. Within the context of the gray bars, “low-risk” and “high-risk” specifically denote noncancer-related risks. “Pts” stands for patients.

Figure 1 captures the pivotal shifts in thoracic surgery [7,8,9,10,11,12]. The color-coded lines delineate the shifting preferences in surgical techniques, from the early practice of pneumonectomy to the advancement of sublobar resections, marking a transition to patient-tailored techniques. This historical overview highlights the move towards preserving lung function and addressing noncancer-related outcomes with consideration to the treatment in non-compromised low-risk patients (above the x-axis in the figure) and compromised high-risk lung cancer patients (below the x-axis). This treatment shift reflects a move away from a one-size-fits-all approach towards a tailored, personalized, precision approach that emphasizes functional preservation despite the increased technical complexity of such surgeries. 

## 3. Milestones in Thoracic Surgery: Evolving from the Early Pneumonectomy to Precision-Based Sublobar Resections

Within this section, we traverse the significant milestones in thoracic surgery, from the first pneumonectomy to the current precise sublobar resections, which underscores a history of innovations aimed at enhancing patient outcomes. Table 1 demonstrates evolutions in thoracic surgical techniques encapsulating the pivotal developments and their impact on the fields.

### 3.1. Pioneering Pneumonectomy: The Milestone That Revolutionized Anatomical Lung Resection

In the early 20th century, the landscape of thoracic surgery underwent a significant transformation, largely due to advancements in anesthesia techniques. Innovations such as positive-pressure ventilation and selective one-lung ventilation remarkably reduced intraoperative risks and postoperative complications, revolutionizing the field [13,14]. Among these milestones, the first successful “single-stage” pneumonectomy, performed by Graham, stands as a hallmark in thoracic surgical history [9]. Graham’s groundbreaking procedure, entailing the complete removal of the left lung to address a patient’s central left upper lobe carcinoma [9]. It not only demonstrated the feasibility of a pneumonectomy but also paved the way for more intricate anatomical resections.

Subsequent to Graham’s pioneering work, procedures such as lobectomies, bronchoplasties, chest wall resections, and sublobar segmentectomies transitioned from experimental interventions to routine surgical procedures [11,15,16,17,18,19]. These advancements significantly expanded the surgical equipment, facilitating more personalized and tailored approaches to the treatment of lung cancer.

### 3.2. The Paradigm Shift from Pneumonectomy to Lobectomy: The Dawn of the “Less Is More” Philosophy in Thoracic Surgery

For decades, pneumonectomy was considered the gold standard for lung cancer treatment. Initially, lobectomy was reserved for compromised, high-risk patients. However, the high morbidity and mortality rates associated with pneumonectomies prompted surgeons to explore the effectiveness of lobectomies with systemic mediastinal lymph node dissection, aiming to balance oncological radicality and functional preservation [15].

In 1962, Shimkin demonstrated that lobectomy exhibited similar survival rates to pneumonectomy but with significantly fewer complications [10]. This led to a shift in surgical philosophy towards the “less is more” approach, establishing lobectomy as the new gold standard for lung cancer treatment. The success of lobectomies also spurred investigations into even less extensive sublobar resections, including segmentectomies and wedge resections. Anatomical segmentectomy was initially described by Jensik et al. as a lung cancer treatment [11]. Subsequent studies supported sublobar resections as appropriate alternatives for lung cancer, especially for high-risk patients [20,21]. Over time, some surgeons advocated sublobar resections as appropriate treatment options even for non-compromised patients with early-stage lung cancer [22,23]. 

The theoretical advantages of “lesser” resections include reduced perioperative morbidity and mortality and the potential for further resections for second primary lung cancers in the future based on preserved pulmonary function. However, theoretical disadvantages include a potential increase in local recurrence and a subsequent elevated risk of lung cancer-related death. To address this dilemma, the LCSG821 trial was initiated in 1982 [12].

### 3.3. LCSG821: Lobectomy Remained as the Standard Care for Early-Stage Lung Cancer, and Its Enduring Influence on Thoracic Surgery Practice

The LCSG821 trial aimed to compare sublobar resections with lobectomies for early-stage lung cancer T1 (≤3 cm, N0 non-small cell lung cancer) in its study conducted between 1982 and 1988 [12]. The results indicated that lobectomies offered superior survival rates and lower locoregional recurrence compared with sublobar resections [12]. Although the trial faced significant limitations, including its nature as an equivalency trial designed with a wide equivalence margin (one-sided, *p* = 0.10 as the predefined threshold), a relatively small sample size, inconsistent pre-surgery CT scans, and enrollment of both wedge and sublobar resections, the gold standard of lobectomies for early-stage lung cancer has remained [12].

The impact of LCSG821 extended beyond its immediate findings. It reshaped lung cancer surgery guidelines, predominantly advocating for lobectomies in early-stage lung cancer treatments. This lasting impact on thoracic surgery practices endured until recent large-scale trials in Japan (JCOG0802) [7] and North America (CALGB140503) [8], which emerged following breakthroughs described in the subsequent sections.

### 3.4. Innovations in Minimally Invasive Surgery with Better Noncancer Outcomes, without Compromising Oncological Efficacy

The evolution of MIS, notably through video-assisted thoracic surgery (VATS) and robot-assisted thoracic surgery (RATS), has been pivotal in transforming thoracic surgery, enhancing non-cancer outcomes while preserving the integrity of oncological treatment.

The impact of MIS extends beyond technical aspects. Randomized trials comparing VATS to traditional thoracotomies consistently demonstrate benefits such as reduced postoperative pain, lower morbidity, and decreased inflammatory responses without compromising overall survival rates [24,25,26]. These findings align with nonrandomized studies demonstrating shorter hospital stays, decreased perioperative mortality, and reduced noncancer-related long-term mortality with MIS procedures [6,27,28,29].

The emergence of RATS in thoracic surgery brings additional benefits, including enhanced precision, improved surgeon ergonomics, and 3D visualization, rendering complex procedures such as segmentectomy more accessible and efficient [30]. Studies highlight that RATS is linked to potentially improved lymph nodal dissection (with a larger number of dissected lymph nodes), reduced postoperative complications, and comparable long-term oncological outcomes when compared with VATS and open thoracotomies [31,32,33]. 

These innovations in MIS not only uphold oncological standards but also significantly influence the trajectory of clinical trials by improving noncancer outcomes. The progression in MIS techniques, notably evident in trials such as JCOG0802, might have contributed to the observed safety achievement of zero mortality in the trial, emphasizing the substantial role of MIS in enhancing patient safety and surgical success.

### 3.5. Precise Evaluation and Selection as the Basis for Precision Medicine: Radiological Advancements for Accurate Staging

To conduct successful clinical trials, appropriate study design and meticulous patient selection based on clinical inquiries are imperative. The success of pivotal trials, such as JCOG0802 and CALGB140503, in thoracic surgery owes much to advancements in radiological technologies. These advancements have facilitated more precise patient selection, a crucial element in achieving reliable and generalizable outcomes. Through accurate assessment of tumor characteristics such as size, location, and lymph node involvement, surgeons can identify individuals most likely to benefit from a specific procedure. Additionally, the precise evaluation of anatomical features is essential for ensuring the safety and success of surgeries, particularly in complex procedures such as segmentectomy, where incorrect assessments may directly impact clinical outcomes or trial events.

The development of high-resolution computed tomography (CT) and positron emission tomography (PET) has transformed the diagnosis and treatment of lung cancer. These imaging techniques provide detailed information into tumors and other organs, enhancing the accuracy of lung cancer staging and the assessment of the extent of lung cancer. In the 1980s, during the accrual period of LCSG821 (1982–1988), chest CT scans were emerging as the primary imaging modality for lung cancer staging, although not consistently obtained, while PET began gaining prominence in lung cancer diagnosis in the late 1990s [34].

### 3.6. Sublobar Resection: Precision in Patient Selection Fueling the “Less Is More” Movement in Thoracic Surgery

The landscape of thoracic surgery, particularly concerning early-stage lung cancer, has been significantly shaped by recent randomized prospective studies, namely JCOG0802 and CALGB140503 [7,8]. These landmark studies have highlighted the advantages of sublobar resections, demonstrating outcomes that range from non-inferior to superior when compared with lobectomies. This shift towards less invasive procedures is deeply rooted in the “less is more” philosophy, emphasizing patient-centric care that balances oncological efficacy with the preservation of quality of life. The remarkable successes of JCOG0802 and CALGB140503 largely stem from their meticulous patient selection criteria [7,8]. These trials rigorously evaluated factors such as tumor size, lymph nodal status, and histology both before and during surgery. Such meticulous selection ensured that only the most suitable candidates underwent the respective surgical approaches, a fundamental practice contributing to the trials’ successful outcomes. This underscores the significance of tailored surgical strategies based on individual patient characteristics.

Surgical outcomes for early-stage lung cancer suggest that sublobar resections, including segmentectomies, may offer survival advantages in certain contexts. The JCOG0802 trial reported a 5-year overall survival rate of 94.3% for segmentectomies, surpassing the 91.1% rate for lobectomies. Additionally, segmentectomies exhibited a comparable disease-free survival rate of 88.0% compared to 87.9% for lobectomies [7]. Conversely, the CALGB140503 study indicated a 5-year overall survival rate of 80.3% for sublobar resections versus 78.9% for lobectomies, with disease-free survival rates at 63.6% and 64.1%, respectively [8]. A post hoc analysis of the CALGB140503 trial, scrutinizing sublobar resections (segmentectomies and wedge resections) separately, demonstrated no statistical differences in outcomes: a 5-year overall survival rate of 63.8% for segmentectomies versus 62.5% for wedges; a disease-free survival of 81.9% versus 79.7%, respectively [35]. These findings underscore segmentectomies as a potentially viable option for early-stage lung cancer treatment, offering comparable or even superior long-term survival outcomes compared to lobectomies in certain patient populations.

The JCOG0802 study particularly highlighted that a segmentectomy not only enhanced overall survival across various subgroups but also led to a lower incidence of deaths from noncancer causes, such as respiratory and cerebrovascular diseases, compared with the lobectomy group. This discovery underscores the critical importance of considering noncancer outcomes in surgical decision-making. Similarly, the CALGB140503 study reinforced the notion that sublobar resections—encompassing both segmentectomies and wedge resections—can attain comparable disease-free survival rates to lobectomies without compromising overall survival. This equivalency in oncological outcomes further validates the shift towards less extensive lung resections. Expanding on these findings, the JCOG1211 trial broadens the applicability of a segmentectomy to tumors >2 cm and ≤3 cm, particularly in cases exhibiting a predominance of ground-glass opacities (GGO) [36]. This emphasis on GGO predominance aims to extend the benefits of a segmentectomy to a wider patient demographic, potentially offering improved outcomes for individuals with specific tumor characteristics.

The differential uptake of sublobar resections across different regions, including Europe, North America, and Asia, reflects a complex interplay of factors beyond geographical preferences. For instance, pivotal trials such as JCOG0802 in Japan and CALGB140503 in North America have delineated substantial disparities in long-term survival post-segmentectomy. For instance, JCOG0802 reported a 5-year overall survival rate of 94.3%, contrasting with 81.9% in CALGB140503 [7,35]. Similarly, a 5-year disease-free survival rate of 88.0% in JCOG0802 versus 63.8% in CALGB140503 suggests variations beyond racial differences. These differences in post-lung resection outcomes are primarily attributed to both tumor oncology and patient functionality. Notably, histological variances are evident, with adenocarcinoma accounting for 90.9% of cases in JCOG0802 compared to 67.9% in CALGB140503. Regarding patient functionality, a notable difference exists in performance status, with 98.2% of JCOG0802 patients having a status of zero versus 80.2% in CALGB140503. Furthermore, the prevalence of never-smokers differs markedly between the two trials, with 44.2% in JCOG0802 versus 9.9% in CALGB140503, potentially impacting both oncological and functional outcomes [7,35]. These factors are compounded and influenced by various aspects, such as diverse healthcare policies, screening systems, surgery practices, patient selection criteria, and surgeons’ preferences across regions.

Collectively, these trials signify a paradigm shift in thoracic surgery, embracing a “less is more” philosophy. This shift is not solely about minimizing the scope of surgery; it is deeply rooted in precise patient selection. Through meticulous patient selection based on a comprehensive set of criteria, these trials have established new standards in thoracic surgery, emphasizing the significance of tailored treatment strategies that optimize both cancer-related and noncancer-related outcomes.

### 3.7. Navigating the Success and Challenges of Sublobar Resection: The Role of 3D Image-Based Simulation and Intraoperative Tumor Localization in Precision Lung Resection

As thoracic surgery progresses towards minimally invasive techniques, sublobar resections demand a mastery of precision driven by both anatomy and technology. Transitioning from lobectomy to pulmonary function-sparing sublobar resections presents several challenges, including the technical intricacies of complex segmentectomies, the imperative for accurate assessment of individual anatomy for surgical planning, and the requirement for precise intraoperative tumor localization. These challenges are compounded by the requirement for thorough preoperative staging to assess the risk of occult lymph node metastasis and for postoperative predictive pulmonary function assessments, emphasizing the importance of surgical expertise and specialized training programs.

Advancements in medical imaging have led to the widespread adoption of 3D-CT imaging, significantly enhancing both preoperative planning and intraoperative navigation [37,38]. This technology empowers surgeons to construct detailed 3D models of a patient’s unique anatomy, offering a comprehensive understanding of the tumor’s spatial relationship with the surrounding lung tissue and vascular structures. Such precision proves indispensable for planning complex lung resections, particularly segmentectomies. Segmental volumetric assessment based on individual imaging is also crucial for planning safe surgeries, particularly for functionally compromised patients [39]. These precision approaches facilitate complete tumor removal while ensuring well-calculated functional preservation and safe margin distances, and aids in making informed decisions regarding the preservation or division of hilar structures. This meticulous surgical planning results in more precise procedures and improved patient outcomes.

The success of sublobar resections, particularly in small N0 tumors, relies on accurately identifying anatomical structures and tumor locations during surgery. This precision is crucial for achieving oncological radicality with adequate margins while preserving essential hilar structures and peripheral lung tissues. Balancing oncological effectiveness with functional preservation stands as a fundamental aspect of contemporary thoracic surgery. Occult lymph node metastasis in patients who undergo sublobar resections may indicate suboptimal oncological treatment. Even among carefully selected clinical N0 patients included in recent trials, incidences of occult lymph node metastasis ranged from 5–7% [7,35]. Although a conversion to a lobectomy is feasible if lymph node metastasis is detected intraoperatively, a completion lobectomy may pose technical difficulties in patients found to have occult lymph node metastasis after surgery. Recent studies investigating long-term outcomes in patients with an occult N2 after either a lobectomy or a segmentectomy have shown that the type of surgical procedure, either a lobectomy or a segmentectomy, does not significantly affect survival rates; instead, appropriate adjuvant chemotherapy plays a crucial role [40,41]. Therefore, emphasis should be placed on administering adequate adjuvant treatment based on proper staging to improve patient survival rates, rather than solely relying on completion lobectomies.

Innovations in tumor localization, such as virtual-assisted lung mapping or radio frequency identification technologies, have markedly improved the accuracy of lung resections [42]. These techniques enable surgeons to precisely locate tumors during surgery, especially those that are small, deep-seated, or challenging to detect. This ensures the attainment of adequate surgical margins and reinforces the safety and efficacy of oncological resections.

In summary, the integration of 3D-CT imaging for intricate anatomical modeling, coupled with advanced tumor localization techniques, has significantly elevated the precision and effectiveness of lung resections, particularly in the domain of complex segmentectomies. These technological advancements not only ensure a comprehensive oncological approach but also emphasize the significance of preserving lung function, marking a substantial advancement in the application of precision medicine within thoracic surgery.

## 4. Future Perspectives

As thoracic surgery continues to evolve, the future holds promising avenues for further advancements. The integration of artificial intelligence and machine learning into diagnostic and surgical practices holds the potential to revolutionize patient care, offering more precise and personalized treatment strategies. Additionally, the development of bio-compatible materials and the advent of 3D-printed organs may open new possibilities for surgical interventions, particularly benefiting patients unsuitable for traditional procedures due to high noncancer-related risks.

Another area of burgeoning interest lies in the potential synergy between targeted therapies, immunotherapies, and surgical interventions [43]. This convergence could yield more effective and minimally invasive treatments, significantly reducing both cancer-related and noncancer-related morbidity and mortality. Moving forward, conducting multidisciplinary research that accounts for both oncological and noncancer-related outcomes become imperative. The aim is to optimize patient care, aligning with our ultimate goal of preserving lives.

As treatments become increasingly intricate, the need for advanced surgical expertise rises. Proficient training in complex sublobar resections, particularly in MIS, holds immense importance for developing and maintaining a diverse range of technical and manual skills. These skills encompass fundamental techniques such as grasping, hemostasis, and ligation, in addition to more complex, context-specific procedures that are essential in challenging surgical scenarios. The utilization of robotic platforms in surgery brings benefits such as enhanced visualization and improved dissection capabilities. However, it also poses challenges, notably the absence of tactile sensation, compelling surgeons to heavily rely on visual feedback and indirect cues. Within this evolving landscape, thoracic surgeons face the dual challenge of refining their own skills while also mentoring and training the next generation of surgeons to meet the demands of increasingly complex surgical procedures [44].

## 5. Conclusions

The field of thoracic surgery has witnessed remarkable advancements over the years, evolving from the initial pneumonectomy to today’s precision-driven sublobar resections. These milestones have not only enhanced oncological outcomes but have also begun addressing the often-overlooked noncancer-related risks associated with lung cancer treatment. As surgeons, our primary responsibility is to save lives, a goal achievable only through a comprehensive approach that accounts for both cancer-related and noncancer-related outcomes.

This review has outlined pivotal milestones and technological advancements in thoracic surgery, highlighting their profound impact on surgical practices and patient well-being. It serves as a reminder that while we pursue oncological thoroughness and embrace cutting-edge techniques, our ultimate aim remains saving lives. In the future, a multidisciplinary approach that integrates technological advancements with a focus on holistic patient care will be essential in to improve outcomes and achieve our primary objective.

## Figures and Tables

**Table 1:** Evolution of thoracic surgical techniques: From pneumonectomy to precision sublobar resection.

Year	Milestone	Description	Impact on Surgery
1933	Pioneering Pneumonectomy [9]	The first successful pneumonectomy revolutionized anatomical lung resection, advancing intraoperative management through the implementation of positive-pressure one-lung ventilation.	Established pneumonectomy as standard care for lung cancer, but with high complication risks, leading to exploration of lobectomy as an alternative approach.
1962	Lobectomy vs. Pneumonectomy [10]	Similar survival rates between lobectomy and pneumonectomy were noted, with fewer complications following lobectomy.	Led to lobectomy becoming the standard care for lung cancer, spurring the exploration of sublobar resections for early-stage diseases [11]
1995	LCSG821 Trial Results [12]	This trial revealed improved survival rates after lobectomy compared with sublobar resection, necessitating a standard care approach for advancing early-stage lung cancer treatment.	Confirmed lobectomy as the standard care for early-stage lung cancer and prompted exploration of sublobar resections for selected patients.
Late 1990s	MIS Innovations	Introduction of MIS techniques to mitigate the high postoperative complications after traditional open thoracotomy.	Marked a significant shift from thoracotomy to MIS, necessitating surgeons to acquire MIS skills.
Late 1990s	Advances in Preoperative Staging	Innovations in preoperative staging and anatomical assessments driven by advancements in medical technologies (CT, PET, etc.).	Improved accuracy in preoperative staging and anatomical assessment, facilitating imaging for simulation and navigation surgery.
2023	JCOG0802 and CALGB140503 Trial Results [7,8]	Demonstrated superior overall survival with segmentectomy (JCOG0802) and comparable disease-free survival with sublobar resection (CALGB140503) compared with lobectomy in treating peripheral, early-stage lung cancer.	Established sublobar resection as a viable standard of care for appropriately selected patients, emphasizing the need for thoracic surgeons to refine patient selection criteria and master sublobar resection techniques.
Recent	Precision Lung Resection	Development of precision lung resection techniques based on advancements in 3D image-based simulation and intraoperative tumor localization.	Led to the prevalence of precision lung resection, emphasizing the need for surgeons to acquire skills and proficiency in this field.

CT, computed tomography; MIS, minimally invasive surgery; PET, positron emission tomography; 3D, three dimensional.

## References

[1] Nilsson J., Berglund A., Bergström S., Bergqvist M., Lambe M. (2017). The role of comorbidity in the management and prognosis in nonsmall cell lung cancer: A population-based study. Acta Oncol..

[2] Groth S.S., Rueth N.M., Hodges J.S., Habermann E.B., Andrade R.S., D’Cunha J., Maddaus M.A. (2010). Conditional cancer-specific versus cardiovascular-specific survival after lobectomy for stage I non-small cell lung cancer. Ann. Thorac. Surg..

[3] Janssen-Heijnen M.L., Houterman S., Lemmens V.E., Louwman M.W., Maas H.A., Coebergh J.W. (2005). Prognostic impact of increasing age and co-morbidity in cancer patients: A population-based approach. Crit. Rev. Oncol. Hematol..

[4] Janssen-Heijnen M.L.G., van Erning F.N., De Ruysscher D.K., Coebergh J.W.W., Groen H.J.M. (2015). Variation in causes of death in patients with non-small cell lung cancer according to stage and time since diagnosis. Ann. Oncol..

[5] Eguchi T., Bains S., Lee M.C., Tan K.S., Hristov B., Buitrago D.H., Bains M.S., Downey R.J., Huang J., Isbell J.M. (2017). Impact of Increasing Age on Cause-Specific Mortality and Morbidity in Patients with Stage I Non-Small-Cell Lung Cancer: A Competing Risks Analysis. J. Clin. Oncol..

[6] Hristov B., Eguchi T., Bains S., Dycoco J., Tan K.S., Isbell J.M., Park B.J., Jones D.R., Adusumilli P.S. (2019). Minimally Invasive Lobectomy Is Associated with Lower Noncancer-specific Mortality in Elderly Patients: A Propensity Score Matched Competing Risks Analysis. Ann. Surg..

[7] Saji H., Okada M., Tsuboi M., Nakajima R., Suzuki K., Aokage K., Aoki T., Okami J., Yoshino I., Ito H. (2022). Segmentectomy versus lobectomy in small-sized peripheral non-small-cell lung cancer (JCOG0802/WJOG4607L): A multicentre, open-label, phase 3, randomised, controlled, non-inferiority trial. Lancet.

[8] Altorki N., Wang X., Kozono D., Watt C., Landrenau R., Wigle D., Port J., Jones D.R., Conti M., Ashrafi A.S. (2023). Lobar or Sublobar Resection for Peripheral Stage IA Non-Small-Cell Lung Cancer. N. Engl. J. Med..

[9] Graham E.A. (1949). The first total pneumonectomy. Tex. Cancer Bull..

[10] Shimkin M.B., Connelly R.R., Marcus S.C., Cutler S.J. (1962). Pneumonectomy and lobectomy in bronchogenic carcinoma. A comparison of end results of the Overholt and Ochsner clinics. J. Thorac. Cardiovasc. Surg..

[11] Jensik R.J., Faber L.P., Milloy F.J., Monson D.O. (1973). Segmental resection for lung cancer. A fifteen-year experience. J. Thorac. Cardiovasc. Surg..

[12] Ginsberg R.J., Rubinstein L.V. (1995). Randomized trial of lobectomy versus limited resection for T1 N0 non-small cell lung cancer. Lung Cancer Study Group. Ann. Thorac. Surg..

[13] Naef A.P. (1987). Development of thoracic surgery from 1880 to the present. Rev. Med. Suisse Romande.

[14] Brodsky J.B., Lemmens H.J. (2007). The history of anesthesia for thoracic surgery. Minerva Anestesiol..

[15] Cahan W.G. (1960). Radical lobectomy. J. Thorac. Cardiovasc. Surg..

[16] Paulson D.L., Shaw R.R. (1955). Bronchial anastomosis and bronchoplastic procedures in the interest of preservation of lung tissue. J. Thorac. Surg..

[17] Grillo H.C., Bendixen H.H., Gephart T. (1963). Resection of the carina and lower trachea. Ann. Surg..

[18] Coleman F.P. (1947). Primary Carcinoma of the Lung, with Invasion of the Ribs: Pneumonectomy and Simultaneous Block Resection of the Chest Wall. Ann. Surg..

[19] Churchill E.D., Belsey R. (1939). Segmental pneumonectomy in bronchiectasis: The lingula segment of the left upper lobe. Ann. Surg..

[20] Bennett W.F., Smith R.A. (1979). Segmental resection for bronchogenic carcinoma: A surgical alternative for the compromised patient. Ann. Thorac. Surg..

[21] Errett L.E., Wilson J., Chiu R.C., Munro D.D. (1985). Wedge resection as an alternative procedure for peripheral bronchogenic carcinoma in poor-risk patients. J. Thorac. Cardiovasc. Surg..

[22] Pastorino U., Valente M., Bedini V., Infante M., Tavecchio L., Ravasi G. (1991). Limited resection for Stage I lung cancer. Eur. J. Surg. Oncol..

[23] Read R.C., Yoder G., Schaeffer R.C. (1990). Survival after conservative resection for T1 N0 M0 non-small cell lung cancer. Ann. Thorac. Surg..

[24] Bendixen M., Jørgensen O.D., Kronborg C., Andersen C., Licht P.B. (2016). Postoperative pain and quality of life after lobectomy via video-assisted thoracoscopic surgery or anterolateral thoracotomy for early stage lung cancer: A randomised controlled trial. Lancet Oncol..

[25] Kirby T.J., Mack M.J., Landreneau R.J., Rice T.W. (1995). Lobectomy—Video-assisted thoracic surgery versus muscle-sparing thoracotomy. A randomized trial. J. Thorac. Cardiovasc. Surg..

[26] Sugi K., Kaneda Y., Esato K. (2000). Video-assisted thoracoscopic lobectomy achieves a satisfactory long-term prognosis in patients with clinical stage IA lung cancer. World J. Surg..

[27] Boffa D.J., Dhamija A., Kosinski A.S., Kim A.W., Detterbeck F.C., Mitchell J.D., Onaitis M.W., Paul S. (2014). Fewer complications result from a video-assisted approach to anatomic resection of clinical stage I lung cancer. J. Thorac. Cardiovasc. Surg..

[28] Paul S., Altorki N.K., Sheng S., Lee P.C., Harpole D.H., Onaitis M.W., Stiles B.M., Port J.L., D’Amico T.A. (2010). Thoracoscopic lobectomy is associated with lower morbidity than open lobectomy: A propensity-matched analysis from the STS database. J. Thorac. Cardiovasc. Surg..

[29] Nwogu C.E., D’Cunha J., Pang H., Gu L., Wang X., Richards W.G., Veit L.J., Demmy T.L., Sugarbaker D.J., Kohman L.J. (2015). VATS lobectomy has better perioperative outcomes than open lobectomy: CALGB 31001, an ancillary analysis of CALGB 140202 (Alliance). Ann. Thorac. Surg..

[30] Eguchi T., Miura K., Hamanaka K., Shimizu K. (2022). Robotic segmentectomy using a lung base-flip approach. JTCVS Tech..

[31] Cerfolio R.J., Bryant A.S., Skylizard L., Minnich D.J. (2011). Initial consecutive experience of completely portal robotic pulmonary resection with 4 arms. J. Thorac. Cardiovasc. Surg..

[32] Park B.J., Melfi F., Mussi A., Maisonneuve P., Spaggiari L., Da Silva R.K., Veronesi G. (2012). Robotic lobectomy for non-small cell lung cancer (NSCLC): Long-term oncologic results. J. Thorac. Cardiovasc. Surg..

[33] Yang H.X., Woo K.M., Sima C.S., Bains M.S., Adusumilli P.S., Huang J., Finley D.J., Rizk N.P., Rusch V.W., Jones D.R. (2017). Long-term Survival Based on the Surgical Approach to Lobectomy For Clinical Stage I Nonsmall Cell Lung Cancer: Comparison of Robotic, Video-assisted Thoracic Surgery, and Thoracotomy Lobectomy. Ann. Surg..

[34] Schrevens L., Lorent N., Dooms C., Vansteenkiste J. (2004). The role of PET scan in diagnosis, staging, and management of non-small cell lung cancer. Oncologist.

[35] Altorki N., Wang X., Damman B., Mentlick J., Landreneau R., Wigle D., Jones D.R., Conti M., Ashrafi A.S., Liberman M. (2024). Lobectomy, segmentectomy, or wedge resection for peripheral clinical T1aN0 non-small cell lung cancer: A post hoc analysis of CALGB 140503 (Alliance). J. Thorac. Cardiovasc. Surg..

[36] Aokage K., Suzuki K., Saji H., Wakabayashi M., Kataoka T., Sekino Y., Fukuda H., Endo M., Hattori A., Mimae T. (2023). Segmentectomy for ground-glass-dominant lung cancer with a tumour diameter of 3 cm or less including ground-glass opacity (JCOG1211): A multicentre, single-arm, confirmatory, phase 3 trial. Lancet Respir. Med..

[37] Nakazawa S., Shimizu K., Kawatani N., Obayashi K., Ohtaki Y., Nagashima T., Eguchi T., Yajima T., Shirabe K. (2020). Right upper lobe segmentectomy guided by simplified anatomic models. JTCVS Tech..

[38] Shimizu K., Nakazawa S., Nagashima T., Kuwano H., Mogi A. (2017). 3D-CT anatomy for VATS segmentectomy. J. Vis. Surg..

[39] Miura K., Eguchi T., Ide S., Mishima S., Matsuoka S., Takeda T., Hamanaka K., Shimizu K. (2023). Bronchial branching patterns and volumetry in the right upper lobe: Impact on segmentectomy planning. Interdiscip. Cardiovasc. Thorac. Surg..

[40] Hui W.K., Charaf Z., Hendriks J.M.H., Van Schil P.E. (2023). True Prevalence of Unforeseen N2 Disease in NSCLC: A Systematic Review + Meta-Analysis. Cancers.

[41] Liou D.Z., Chan M., Bhandari P., Lui N.S., Backhus L.M., Shrager J.B., Berry M.F. (2022). Lobar versus sublobar resection in clinical stage IA primary lung cancer with occult N2 disease. Eur. J. Cardiothorac. Surg..

[42] Sato M. (2020). Precise sublobar lung resection for small pulmonary nodules: Localization and beyond. Gen. Thorac. Cardiovasc. Surg..

[43] Forde P.M., Spicer J., Lu S., Provencio M., Mitsudomi T., Awad M.M., Felip E., Broderick S.R., Brahmer J.R., Swanson S.J. (2022). Neoadjuvant Nivolumab plus Chemotherapy in Resectable Lung Cancer. N. Engl. J. Med..

[44] Eguchi T., Shimura M., MIshima S., Hara D., Matsuoka S., Kumeda H., Miura K., Hamanaka K., Shimizu K. (2023). Tailored Practical Simulation Training in Robotic Surgery: A New Educational Technology. Ann. Thorac. Surg. Short. Rep..

